# Transcriptional profiling of interleukin-2-primed human adipose derived mesenchymal stem cells revealed dramatic changes in stem cells response imposed by replicative senescence

**DOI:** 10.18632/oncotarget.4852

**Published:** 2015-07-14

**Authors:** Ping Niu, Aibek Smagul, Lu Wang, Aiman Sadvakas, Ying Sha, Laura M. Pérez, Aliya Nussupbekova, Aday Amirbekov, Akan A. Akanov, Beatriz G. Gálvez, I. King Jordan, Victoria V. Lunyak

**Affiliations:** ^1^ Department of Pediatrics, Renmin Hospital of Wuhan University, Wuhan, China; ^2^ S.D. Asfendiyarov Kazakh National Medical University, Almaty, Kazakhstan; ^3^ School of Biology, Georgia Institute of Technology, Atlanta, GA, USA; ^4^ Cardiac Development and Repair Department, National Center for Cardiovascular Research (CNIC), Madrid, Spain; ^5^ PanAmerican Bioinformatics Institute, Santa Marta, Magdalena, Colombia; ^6^ Aelan Cell Technologies, Inc, San Francisco, CA

**Keywords:** mesenchymal stem cells, IL-2, aging, cancer, immunomodulation

## Abstract

Inflammation is a double-edged sword with both detrimental and beneficial consequences. Understanding of the mechanisms of crosstalk between the inflammatory milieu and human adult mesenchymal stem cells is an important basis for clinical efforts. Here, we investigate changes in the transcriptional response of human adipose-derived stem cells to physiologically relevant levels of IL-2 (IL-2 priming) upon replicative senescence. Our data suggest that replicative senescence might dramatically impede human mesenchymal stem cell (MSC) function *via* global transcriptional deregulation in response to IL-2. We uncovered a novel senescence-associated transcriptional signature in human adipose-derived MSCs hADSCs after exposure to pro-inflammatory environment: significant enhancement of the expression of the genes encoding potent growth factors and cytokines with anti-inflammatory and migration-promoting properties, as well as genes encoding angiogenic and anti-apoptotic promoting factors, all of which could participate in the establishment of a unique microenvironment. We observed transcriptional up-regulation of critical components of the nitric oxide synthase pathway (iNOS) in hADSCs upon replicative senescence suggesting, that senescent stem cells can acquire metastasis-promoting properties via stem cell-mediated immunosuppression. Our study highlights the importance of age as a factor when designing cell-based or pharmacological therapies for older patients and predicts measurable biomarkers characteristic of an environment that is conducive to cancer cells invasiveness and metastasis.

## INTRODUCTION

Transplanted mesenchymal stem cells (MSCs) are consistently exposed to tissue signals, immune cells and mediators that could influence their behavior. The mechanisms by which this environment influences potential therapeutic outcomes in MSC clinical applications remain poorly understood, however, over the past decade MSCs themselves have been shown to possess a broad spectrum of signaling capabilities, affecting both adaptive and innate immunity by the secretion of growth factors and chemokines to induce cell proliferation, angiogenesis, interactions with the immune system and mediation of anti-apoptotic events [1, 2]. Based on these remarkable properties, the MSCs are considered to have therapeutic potential to treat broad spectrum of diverse human diseases, including cancer [3].

The most favorable way of using the full potential of MSCs as a therapeutic is the clinical utilization of autologous (patient specific) or syngeneic (genetically similar) cells. To date, little is known regarding the extent to which the beneficial properties of MSC change with the age of the patient or upon MSCs expansion and passaging *ex-vivo,* where the length of expansion period, culture methods and the patient's clinical history can all lead to a gradual accumulation of replicatively senescent cells. Replicative senescence is characterized by a growth arrest, apoptosis resistance, morphological and cell-size changes, high levels of expression of the tumor suppressors *P16*, *P21*, *P53* and/or *RB*, increased activity of senescence-associated *beta* galactosidase (SA-*β*-gal) and loss of the ability to synthesize and repair DNA [4, 5]. Numerous reports indicate that the replicative aging of stem cells and MSCs, in particular, can influence their biological properties, including their ability to secrete beneficial factors [6-9].

The primary trophic property of MSCs is secretion of mitogenic growth factors such as transforming growth factor-alpha (TGF-α), TGF-β, hepatocyte growth factor (HGF), epithelial growth factor (EGF), basic fibroblast growth factor (FGF-2), vascular endothelial growth factor (VEGF) and insulin-like growth factor-1 (IGF-1). All of these factors, when present in the systemic milieu, have shown to increase fibroblasts along with epithelial and endothelial cell division or differentiation [3, 10-13]. This provides evidence that the MSC-triggered cellular communication circuitry is necessary for tissue or organ remodeling and regeneration. Interestingly, the secretion of a wide array of growth factor and anti-inflammatory proteins by MSCs could also be modulated in response to inflammatory molecules, such as interlukin-1 (IL-1), IL-2, IL-12, tumor necrosis factor-alpha (TNF-α) and interferon-gamma (INF-γ) (see for review [3]), thereby providing complex signaling guidance to many inflammatory cells, including T-cells, natural killer cell, B-cells, monocytes, macrophages and endritic cells [3, 14-17]. Previous reports have demonstrated that pro-inflammatory cytokines were able to increase the migration of human MSCs as well as to induce the production of chemokines and chemotactic factors that permit MSCs to suppress immune reactions [18-20]. The best documented immune-modulatory effect of MSCs is their ability to impose G0/G1 phase arrest in the activated T-cells, thus inhibiting T-cell proliferation [19, 20] [21, 22]. Despite the notion that the secretion by MSCs of a number of the soluble factors (IL-6, IL-10, indoleamine, 2,3-dioxygenase, iNOS and PGE2) could assist injury through the modulation of the regenerative environment *via* anti-inflammatory and immunomodulatory pathways, the exact molecular mechanism by which this modulation takes place is only partially understood and seemingly contradictive, in part due to lack of data that clearly articulates how adult stem cell aging *in-vivo* or *ex-vivo* contributes to immunomodulatory properties.

This study was conducted to evaluate the impact of replicative senescence on the transcriptional activity of human adipose-derived MSCs (hADSCs) in response to IL-2 signaling. Our results uncovered significant changes imposed by replicative senescence on biological pathways related to stem cell response to IL-2 priming, and suggest that such changes might dramatically influence outcomes of clinical application of hADSCs by affecting their immunomodulatory and migration properties as well as their ability to influence the regenerative environment.

## RESULTS

### Characterization of the MSC senescent phenotype

**Figure 1:**
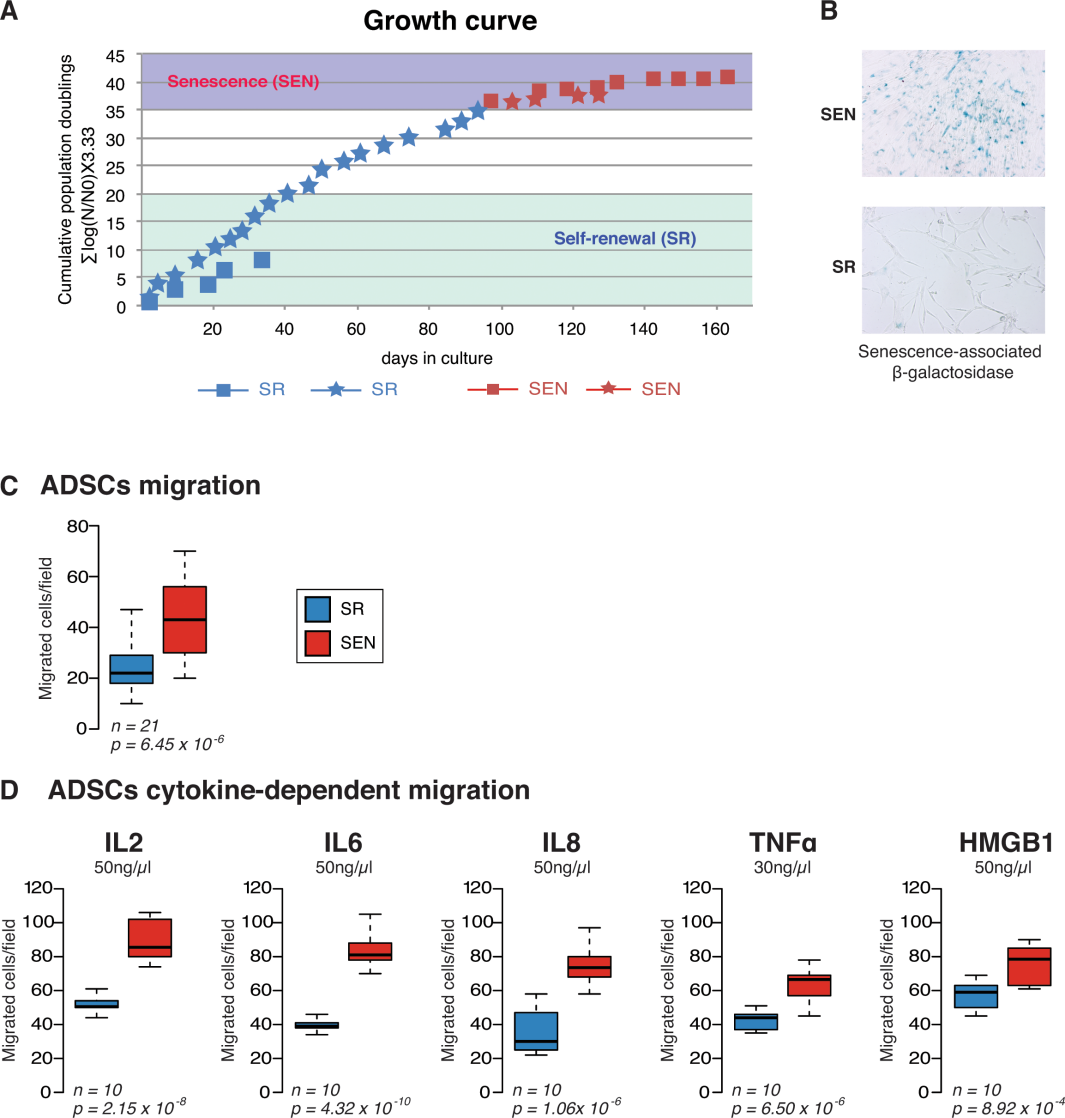
Replicative senescence impairs migratory properties of the hADSCs **A.** Growth curve of the hADSCs is represented as cumulative population doubling over day in culture. Two patient-specific cell lines were used for the study (Female, 32 years old, *-Female 45 years old). Linear proliferation stage for both lines shown in blue (SR) and stages when hADSC enter senescence shown in red (SEN) **B.** Colometric detection of senescence-associated β-galactosidase (10x) in self-renewing (SR) and senescent (SEN) hADSCs. **C.** *Ex-vivo* migration assays for self-renewing (SR-blue) and senescent (SEN-red) hADSCs were performed in complete DMEM-F12 media. The black lines indicate the median values, and the whiskers indicate the range of values. Statistical difference was evaluated by *t*- test with *P-*value (*p*) as depicted. **D.** Self-renewing SR (blue) and senescence SEN (red) hADSCs were induced to migrate in the presence of different cytokines (50 ng/ml IL-2, IL-6, IL-8, HMGB1; 30 ng/ml TNF). The graphic represents the mean of ten independent experiments (n = 10). *P*-values (p) related to experimental measurements are listed under the graphs.

Mesenchymal stem cells (MSCs) are mesoderm-derived cells that reside in the stroma of solid organs and function as precursors of non-hematopoietic connective tissues with the capacity to differentiate into mesenchymal and non-mesenchymal cell lineages. Adipose-derived MSCs (ADSCs) are more accessible, compared to bone marrow BM-MSCs, more abundant, and equally capable of differentiating into cells and tissues of mesodermal origin [23]. ADSCs also share some of the immunomodulatory properties that characterize BM-MSCs. Reported data indicate that ADSCs could effectively down-regulate excessive immunologic reactions and have a protective effect on acute graft-versus-host disease, as well as in animal models of experimental arthritis [24, 25]. hADSCs were isolated and cultured as described in the Materials and Methods and in [7]. *Ex-vivo* replicative senescence led to decreased proliferation, accumulation of DNA damage and observed typical morphological changes: hADSCs became much larger with irregular and flat shape, and nuclei became more circumscribed in phase contrast microscopy with the granular cytoplasm appearance of many inclusions and aggradations [6, 7]. The growth curves of hADSCs obtained from two different patients are shown in Figure 1A. Typical staining for senescence-associated SA-β galactosidase activity for either hADSCs in linear growth rate, self-renewing state (SR) or when cell lines cease their proliferation, senescent state (SEN) is shown in Figure 1B and described in detail in [7].

### Replicative senescence ADSCs demonstrate a higher propensity for migration

One of the important characteristics of MSCs is their ability to migrate to sites of damaged tissue [26]. To investigate whether or not *ex-vivo* replicative senescence affects the migratory potential of hADSCs, we have performed migration assays with a set of common cytokines and relevant growth factors using the Transwell system as described in the Materials and Methods. We observed that SEN hADSCs showed significantly higher basal migration capacity than their SR counterparts (Figure 1C). In addition, the response of SEN hADSCs to different cytokine chemo-attractants was measured. The factors IL-2, IL-6, IL-8 as well as TNF-α and HMGB1 have been previously reported as potent chemo-attractants inducing migration of different stem cell types [27, 28]. Our data indicate that hADSCs at late passages have an increased ability to migrate in comparison to early passages (Figure 1D), indicating that replicative senescence increases the migratory properties of hADSCs in response to the tested chemo-attractants. Interestingly, upon senescence of hADSCs interleukin-2 (IL-2) became the most potent chemo-taxis stimulant whereas the TNF-α is less potent among the tested chemo-attractants in these experiments (Figure 1D).

Collectively, these data indicate that replicative senescence can modify the migratory properties of hADSCs and may possibly influence hADSCs response to the inflammatory environment as well as their immunomodulation output *ex-vivo*.

### Differential response to IL-2 stimulation in human adipose-derived MSCs upon replicative senescence

**Figure 2:**
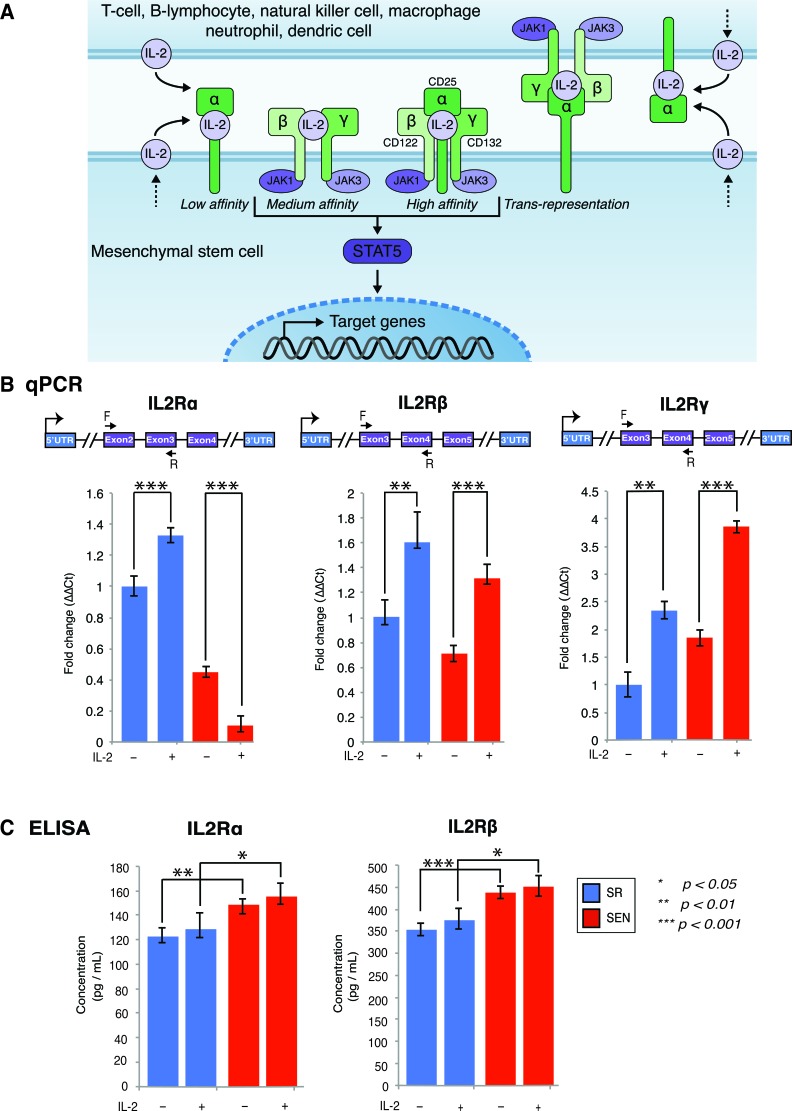
Gene expression of IL-2 receptor isoforms and their association with membrane in self-renewing (SR) and senescent (SEN) hADSCs primed with IL-2 **A.** Three classes of IL-2 receptors (high-affinity, intermediate affinity, and low affinity), with receptor composition and associated JAK kinases are shown as cartoon. Trans-presentation of IL-2Rα expressed by MSCs to immune cells that expresses IL-2Rβ and IL-2Rγ has been depicted separately. The soluble IL-2 receptor (soluble sIL-2Rα) with bound IL-2 is not shown in the figure. **B.** IL-2 receptors α, β, γ assessed by quantitative PCR in un-stimulated (IL-2-) senescent (red) and self-renewing (blue) hADSCs and upon stimulation with 20ug/ml of recombinant IL-2 (IL-2+). Data shown as fold change ΔΔCT Mean +SD from three independent experiments is show. Position of the q-PCR primers off line are depicted graphically **C.** Cellular membrane associated levels of IL-2Rα and IL-2Rβ were quantified by ELISA in un-stimulated (IL-2-) senescent (SEN-red) and self-renewing (SR-blue) hADSCs and upon stimulation with 20ug/ml of recombinant IL-2(IL-2+). Data are expressed as picogram per milliliter. Results are the mean of three independent experiments (mean ± SD). Statistical significance was estimated by *t* - test, where ***p < 0.001, **p < 0.01, *p < 0.05.

IL-2 is a ligand used by cells expressing either the intermediate-affinity receptor dimer of IL2Rβ (CD122) and the common IL2Rγ (CD132), or the high-affinity trimeric IL2R comprising IL2Rα (CD25) in addition to the IL2Rβ and IL2Rγ isoforms (shown in Figure 2A). The intermediate-affinity IL-2 receptor is more broadly expressed on T-cells, natural killer cells and monocytes [29]. The high-affinity IL-2 receptor is constitutively expressed on regulatory T-cells (T_reg_). Information about the role of IL-2 signaling in non-T-cells is limited, but nevertheless suggests the existence of similar stimulatory receptor representations in other cell types [30-32]. Currently, it is unknown how hADSCs are affected by therapeutic doses of IL-2, and whether or not there are changes that occur in IL-2 receptor composition or IL-2 receptor signaling upon *ex-vivo* replicative senescence of this type of human MSCs.

Assessment of the IL-2 receptor isoforms expression by qPCR demonstrated dramatic changes in expression of the *IL2R*α isoform in comparison to *IL2R*γ and *IL2R*β upon replicative senescence *ex-vivo* (Figure 2B). Notably, the increased accumulation of the *IL2R*β and *IL2R*γ transcripts was recorded after IL-2 treatment in both SR and SEN hADSCs, whereas *IL2R*α expression was severely abrogated when senescent cells were subjected to similar treatments (Figure 2B). However, our data indicate that on the protein level, the cellular membrane-associated IL2Rα receptor shows the opposite pattern (Figure 2C). Although the transcriptional status of IL-2 receptor isoforms does vary between the two different cell states (SR and SEN), it does not seem to be dependent upon IL-2 priming as measured by the ELISA assay (described in the Materials and Methods). Interestingly, our data also demonstrate that protein encoding IL-2 receptor α chain is far less abundant than the IL2Rβ isoform (compare 120 pg/ml of IL2Rα and 350 pg/ml IL2Rβ to 150 pg/ml of IL2Rα and 440 pg/ml IL2Rβ upon replicative senescence *ex-vivo*) as shown in Figure 2C. These data suggest that hADSCs response to IL-2 stimulation occurs, by and large, through the intermediate-affinity receptor dimer composed of IL2Rβ (CD122) and the common IL2Rγ (CD132). These data also caution against over-interpretation of receptor-signaling pathway dynamics based on transcriptional assays only.

**Figure 3:**
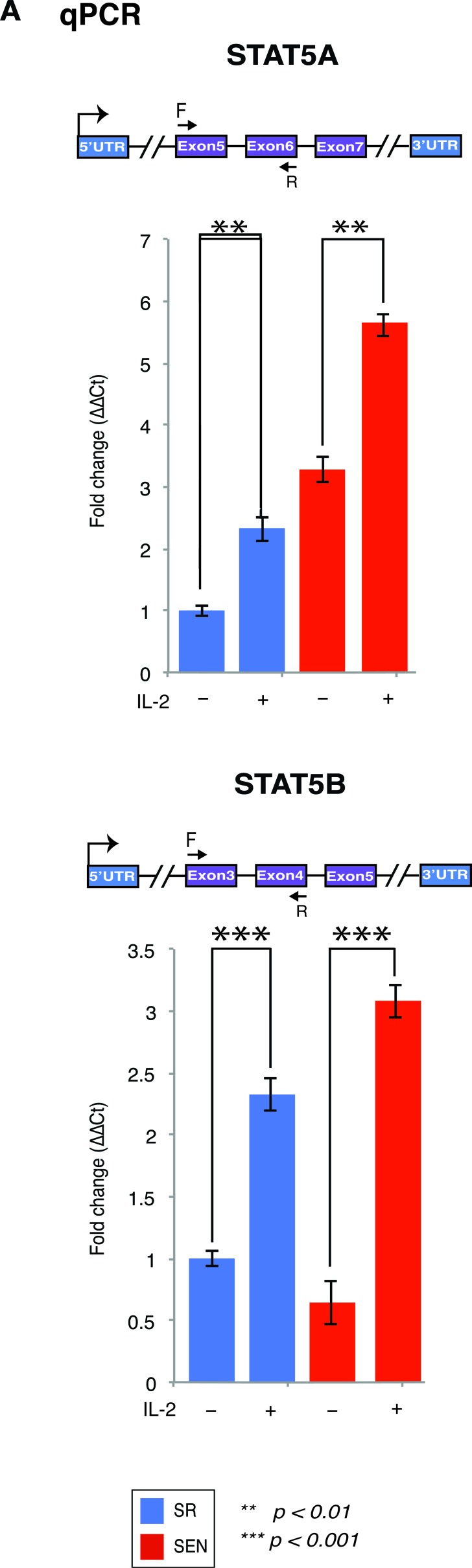
Stimulation of the self-renewing and senescent hADSCs with IL-2 upregulates mRNA of a mediator of IL-2 signaling STAT5 gene *STAT5A* and *STAT5B* mRNA expression was assessed by quantitative RT-PCR in un-stimulated (IL-2-) senescent (red) and self-renewing (blue) hADSCs and upon stimulation with 20ug/ml of recombinant IL-2 (IL-2+). Data shown as fold change ΔΔCT Mean ± SD from three independent experiments is shown. Position of the qPCR primers are depicted graphically. Statistical significance was estimated by *t* - test, where ***p < 0.001, **p < 0.01.

IL-2 signals *via* JAK1 and JAK3 to activate STAT5A and STAT5B, and additionally uses Ras-MAP kinase and phosphoinositol 3-kinase dependent signaling pathways (Figure 2A) [33, 34]. The expression of downstream target of IL-2, STAT5, is shown in Figure 3A and 3B and Supplementary Table 1. In hADSCs, both *STAT5A* and *STAT5B* gene transcription follows the IL-2/STAT5 signaling axis previously described for T-cells (for review see [35]), thus prompting us to investigate in detail how IL-2 and its downstream target STAT5 may effect transcriptional outcomes in hADSCs upon their replicative senescence *ex-vivo*.

### Priming with IL-2 results in altered gene expression in human ADSCs upon replicative senescence

To address how the transcriptional response to the IL-2/STAT5 axis changes upon replicative aging of hADSCs *ex-vivo,* we performed RNA-seq transcriptome analysis using the Ion Proton^TM^ System as described in the Material and Methods and shown in Supplementary Figure 1A. We compared gene expression levels in hADSCs across four conditions (libraries): self-renewal upon normal *ex-vivo* culture (SR IL-2–), self-renewal upon 24 hrs recombinant IL-2 stimulation (SR IL-2+), replicative senescence upon normal *ex-vivo* culture (SEN IL-2–), and replicative senescence upon 24 hrs recombinant IL-2 stimulation (SEN IL-2+). Distributions of the total read counts for the four libraries representing each condition are shown in Supplementary Figure 1B and 1C.

**Figure 4:**
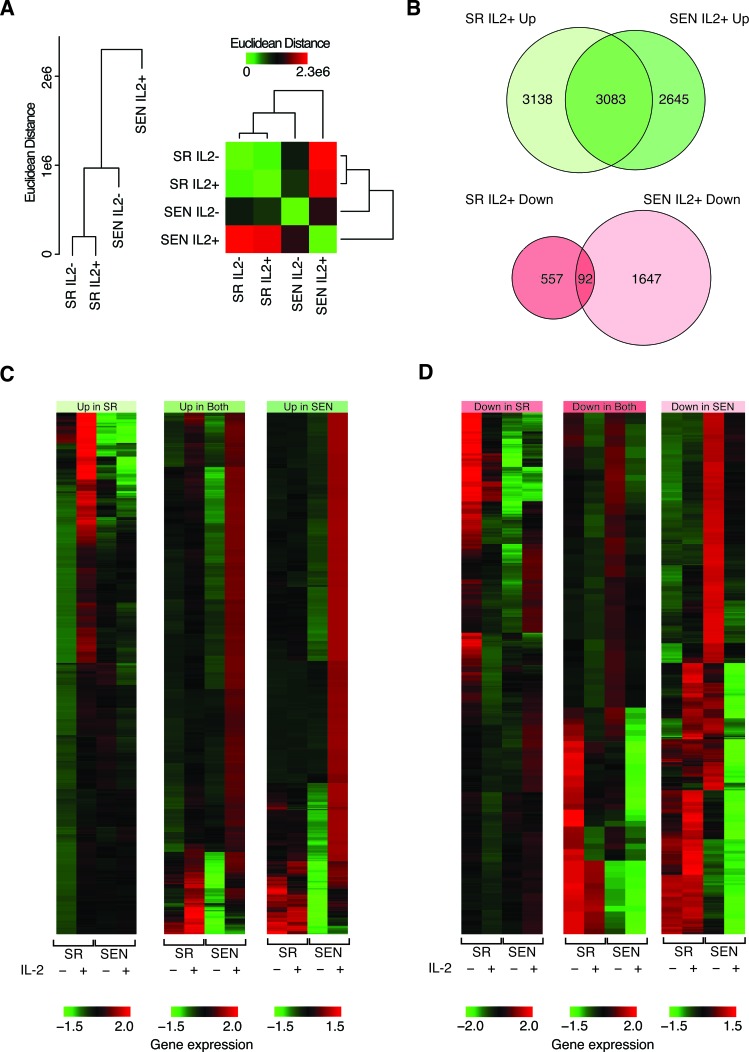
Comparison of gene expression levels between self-renewing and senescence hADSCs upon IL-2 treatment **A.** Hierarchical clustering showing the pairwise distance between conditions based on comparison of condition-specific gene expression profiles. **B.** Venn diagram showing the numbers of genes, which are up-regulated and down-regulated upon IL-2 treatment. **C.** & **D.** Heatmap showing the expression levels of genes that are up-regulated **C.** and down-regulated **D.** upon IL-2 treatment. Normalized gene expression levels are color coded as shown in the legend (red = high & green = low). Groups correspond to genes that are up- or down-regulated in SR-only, SEN-only or both conditions.

We used beta-actin expression levels to normalize gene expression levels between conditions (see Materials and Methods). This approach was taken to allow for the fact that overall gene expression levels are likely to change upon IL-2 treatment, and using a global normalization method to bring the overall expression distributions for each condition into register would remove this biological signal. Indeed, beta-actin normalized gene expression distributions reveal overall up-regulation of gene expression upon IL-2 treatment in both SR and SEN states (Supplementary Figure 1D). However, comparison of individual gene expression levels among the four conditions suggests that IL-2 treatment more dramatically affects senescent (SEN) compared to self-renewing (SR) hADSCs (Figure 4A). The SR IL-2– and SR IL-2+ conditions group closely together when individual gene expression levels are compared followed by the SEN IL-2– condition. The SEN IL-2+ condition is a clear outlier amongst the four conditions showing a substantially divergent pattern of individual gene expression levels. This suggests the possibility that the biological response to IL-2 treatment in hADSCs upon senescence might dramatically impede MSC function *via* global transcriptional de-regulation in response to IL-2.

Expression levels were further compared between conditions in order to identify individual genes that are differentially expressed, up- and down-regulated, in response to IL-2 treatment in both SR and SEN states (Figure 4B). There are far more genes that are up-regulated (8,866) compared to down-regulated (2,296) upon IL-2 treatment in both SR and SEN hADSCs. There is also a substantially higher proportion of genes that are up-regulated in both SR and SEN hADSCs (35%) compared to genes that are down-regulated in both states (4%). The greatest asymmetry is seen for genes that are down regulated in SEN hADSCs upon IL-2 treatment (1,739); there are many more such genes than seen for the SR IL-2+ condition (649). This difference suggests that the overall divergence of the SEN IL-2+ condition is largely attributed to genes that are down-regulated upon IL-2 treatment, which is an unexpected result given the overall up-regulation across both SR and SEN upon IL-2 treatment (Figure 4B, 4C, 4D and Supplementary Figure 1D).

Taken together, these data suggest the possibility that SEN hADSCs have lost the ability to generate a response to IL-2 treatment to the same extent as actively proliferating cells. The greater number of up-regulated genes seen for SR IL-2+, compared to SEN IL-2+, is consistent with this interpretation.

### Trophic properties of the hADSCs after interleukin-2 priming are susceptible to replicative aging *ex-vivo*

**Figure 5:**
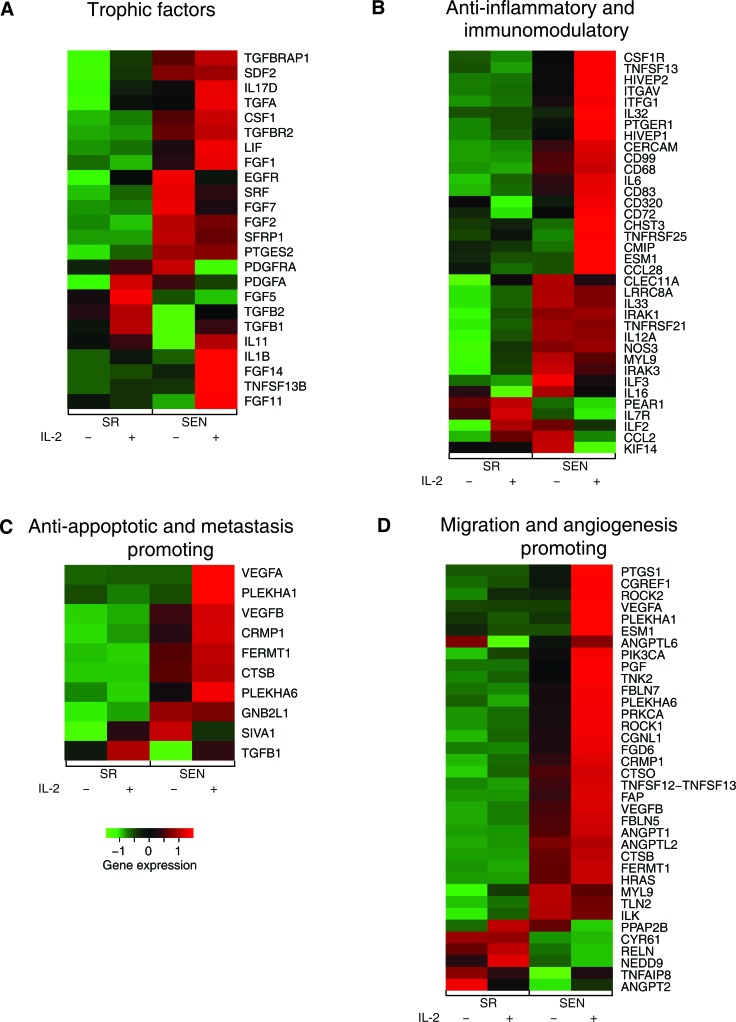
Gene expression levels for self-renewing and senescence hADSCs upon IL-2 treatment among functionally coherent sets of genes Expression levels are shown for sets of genes characterized as **A-** trophic factors, **B-** anti-inflammatory and immunomodulatory, **C-** anti-apoptotic and metastasis promoting, and **D.** migration and angiogenesis promoting. Normalized gene expression levels are color coded as shown in the legend (red = high & green = low).

The secretion of a broad range of bioactive molecules is now believed to be the main mechanism by which MSCs achieve their therapeutic effects. MSCs secrete an array of growth factors and anti-inflammatory proteins with complex feedback mechanisms among the many types of immune cells [3]. Our data indicate that the expression of growth factors in hADSCs upon stimulation with IL-2 is subjected to dramatic changes upon replicative senescence *ex-vivo*. While the priming of actively proliferating (SR) hADSCs with IL-2 results in increased expression of mitogenic proteins such as stromal cell-derived factor-2 (*SDF2*) and *SDFL2*, and prostaglandin E synthetase-2 (*PTGES2*), both SR and SEN ADSCs are marked by drastic increases of transforming growth factors *alpha* and *beta* (*TGF*α*, TGF*β*1* and *TGF*β*2*), transforming growth factor *beta* receptor *TGFBR2* and transforming growth factor *beta* receptor-associated protein *TGFBRAP1,* as well as transforming growth factor *beta*-induced (*TGFBI*), which are known to increase fibroblast, epithelial and endothelial cell division when secreted in systemic milieu (Figure 5A and Table S1) [3, 36]. In addition, both SR and SEN IL-2 stimulated hADSCs are marked by up-regulation of colony stimulating factor-1 (*CSF-1*), *LIF*, *IL-11*, *IL-17D*, *IL-1*β and tumor necrosis factor (ligand) superfamily *TNFSF13B*, a cytokine encoding gene that stimulates B- and T-cell function (Figure 5A, 5B and Supplementary Table1). Taking into account that paracrine IL-17D induces expression of *IL-6*, *IL-8*, and *GM-CSF* genes in endothelial cells, and IL-1β stimulates fibroblast growth factor activity (*TGF*α*, TGF*β*1* and *TGF*β*2* genes are notably up-regulated in IL-2-primed hADSCs) in autocrine and paracrine fashion, along with thymocyte and B-cell proliferation and maturation by inducing release of IL-2 from these cells, our data suggest that the transcriptional status of both SR and SEN hADSCs may point to enhanced immunomodulatory properties of these cells after IL-2 priming *via* a complex regulatory feed-back loop.

In addition, we also noted essential differences in the IL-2 dependent expression of growth factors upon senescence of hADSCs that have not been observed in SR cells. This includes up-regulation of only a subset of fibroblast growth factor family members (*FGF1, FGF11, FGF14*) accompanied by down-regulation of other members, such as *FGF2, FGF5, FGF7,* (Figure 5A and Supplementary Table1). Surprisingly, IL-2 primed SEN hADSCs are marked by *EGF* mRNA up-regulation, but down-regulation of mRNA to its receptor *EGFR,* together with decrease in expression of the serum response factor *SRF* and the secreted modulator of WNT signaling *SFRP1*. Interestingly, the expression of both a potent mitogen for cells of mesenchymal origin that promotes wound healing, *PDGFA,* and its receptor, *PDGFRA,* is drastically suppressed in SEN hADSCs in comparison to SR cells subjected to IL-2 priming (Figure 5A, Table 1 and Supplementary Table 1).

These data revealed essential senescence-related differences in the nature of IL-2 mediated transcriptional response in hADSCs that might impede their immunomodulatory properties *ex-vivo* and, ultimately, *in-vivo*.

### Anti-inflammatory and immunomodulatory properties of IL-2 primed human MSC

Next, we investigated how exposure to the IL-2 pro-inflammatory environment, when imposed on replicative aging, affects the expression of the genes assigned to provide immunomodulatory properties of hADSCs. Published data indicated that MSCs can prevent proliferation and promote differentiation of many inflammatory immune cells, including T-cells, natural killer cells, B-cells, monocytes, macrophages and dendritic cells through the secretion of paracrine factors in response to inflammatory environment [20]. Our data demonstrate that this capacity for immunomodulation could be severely affected by replicative aging of the human adipose-derived MCS during *ex-viv*o passaging (Figure 5B) and Supplementary Table 1.

IL-2 priming in SEN hADSCs activates distinct set of genes attributed to T-cell regulation. IL-2 priming of self-renewing hADSCs results in up-regulation of genes, such as *TNFRSF21* (involved in T-cells differentiation), *IL12A* (T-cell activator), *ILF2* (potent regulator of transcription of the *IL-2* gene during T-cell activation), *IL33* (paracrine inducer of T-helper type 2 associated cytokines) and down-regulation of *CCL28* (chemotactic factor for CD4+, CD8+ T-cells), *CD320* (receptor molecule with autocrine and paracrine function to augment the proliferation of plasma cells) shown in Figure 5B and Supplementary Table 1. Contrary to that, IL-2 primed senescent hADSCs are characterized by drastic transcriptional up-regulation of *CD320,* a number of integrins which could be involved in modulation of T-cell function (*ITG11*, *ITGAV, ITFG1*), and genes encoding important regulatory molecules such as: the T-cell adhesion receptor (*CD99*), a factor attributed to the maintenance of naïve T-cells (*CHST3*), T-cell activators (*HIVEP1* and *HIVEP2*), a gene involved in T-cell signaling pathway (*CMIP*) and an autocrine/paracrine factor, *PTGER1*, involved in inhibition of CD+ cell proliferation (Figure 5B and Supplementary Table1). Our data also demonstrate that SR hADSCs exposed to IL-2 trigger down-regulation of transcriptional activities of the genes encoding surface receptors that play a role in B-cell proliferation and differentiation (*CD72*) and homing macrophages (*CD68*). Both of these genes are significantly transcriptionally up-regulated in senescent cells upon similar treatment (Figure 5B and Supplementary Table 1).

In addition, IL-2 treated SEN hADSCs are set apart from similarly treated SR cells by transcriptional down-regulation of the genes required for pro-B to pre-B transitioning, the *LRRC8A* and *PEAR1* genes, that regulate a number of non-adherent myeloid progenitors. In contrast, the genes involved in lymphocyte activation and homeostasis (*CD83* and *TNFRSF25*) as well as leukocyte transmigration (*CERCAM*), and the genes responsible for endothelial cell-leukocyte interaction (*ESM1*), and a gene important for the control of monocytes/macrophage mediated immunological process (*TNFSF13)*, are up-regulated in SEN hADSCs (Figure 5B and Supplementary Table 1).

These observations, together with IL-2 dependent differential transcriptional expression of cytokines in SEN hADSCs (up-regulation of *IL-32, IL-6*, *PLAU* genes; down-regulation of *CCL2, CLEC11A, ILF3, IRAK3, KIF14, MYL9* genes) and in SR hADSCs (up-regulation of *IL12A, IL7R, IRAK1, NOS3* genes*;* down-regulation of *IL16, CSF1R* genes), indicate that the putative immunomodulatory properties of hADSCs are susceptible to senescence imposed changes and suggest novel biological pathways and gene targets that can be further explored *ex-vivo* and *in-vivo*.

### Anti-apoptotic and metastasis promoting properties of IL-2-primed hADSCs upon replicative senescence

**Figure 6:**
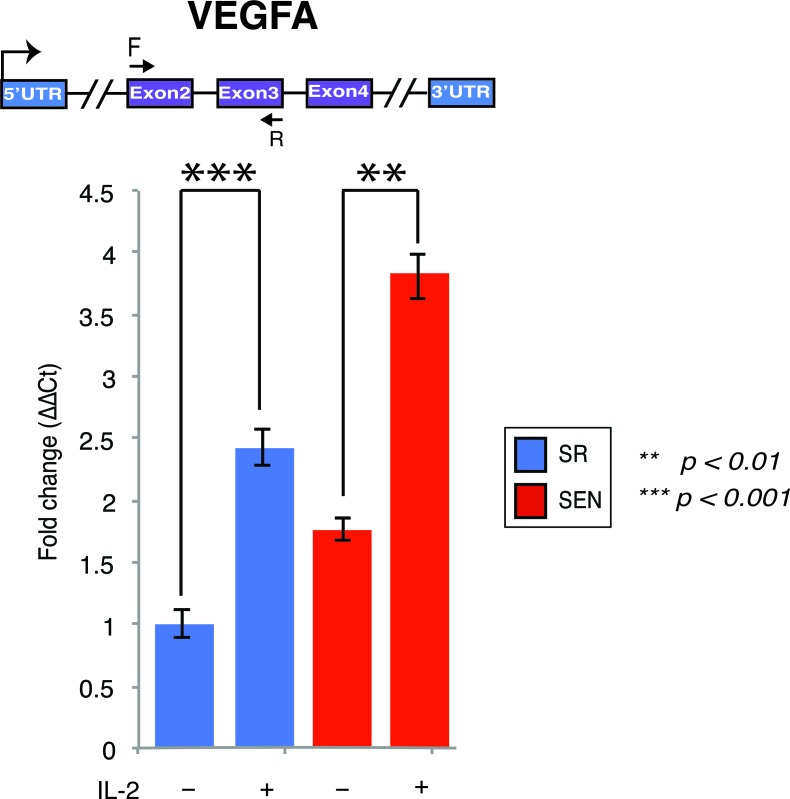
Interleukin-2 upregulates transcription of the VEGFA gene upon replicative senescence of hADSCs *VEFGA* gene expression was assessed by quantitative qPCR in unstimulated (IL-2-) senescent (red) and self-renewing (blue) hADSCs and upon stimulation with 20ug/ml of recombinant IL-2 (IL-2+). Data shown as fold change ΔΔCT Mean ± SD from three independent experiments is show. The position of the q-PCR primers are depicted graphically. Statistical significance was estimated by *t* - test, where ***p < 0.001, **p < 0.01.

Another important clinical property of MSCs is the ability to rescue apoptotic cell death induced by traumatic exposures to hypoxia, chemicals/acidity, mechanical damage and radiation. For example, MSCs have been proven to assist reversal of apoptosis in cardiomyoblasts after ischemia, as well as damaged neurons and lung fibroblasts [37, 38]. Recently, stanniocalcin-1 (STC1) was identified as an essential factor capable of potent apoptotic reversal in fibroblasts damaged by UV and acidity [39]. Our data indicate that IL-2 priming transcriptionally upregulates both *STC1* and *STC2* genes, and such activation is not dependent on the replicative aging of hADSCs, at least *ex-vivo* (Supplementary Table1). In addition, paracrine effectors such as *VEGF* and *TGF*β*1* have been implicated in the reversal of apoptosis in endothelial cells [40]. The expression of genes encoding both of these factors is up-regulated in SR and SEN hADSCs upon IL-2 treatment (Figure 5C, Figure 6 and Supplementary Table 1). However, transcriptional activity of *VEGFA* is notably higher in senescence then in actively proliferating cells as further verified by qPCR analysis shown in Figure 6. Notably, the *SIVA1* gene encoding a pro-apoptotic factor and a potent inducer of T-lymphocytes apoptosis [41] is significantly down-regulated in senescent cells upon IL-2 treatment in comparison to proliferating hADSCs (Figure 5C and Supplementary Table1). In addition, recently published data indicate that SIVA1 is not a strictly pro-apoptotic factor, but also a potent suppressor of tumor metastasis [42]. Importantly, a number of the factors responsible for invasive growth and metastasis are significantly up-regulated in SEN hADSCs primed with IL-2 in comparison with similarly treated SR cells (Figure 5C). This includes *PLEKHA1, PLEKHA6, CTSB, CRMP1, FERMT1* genes.

These data indicate that pre-treatment/priming of hADSCs with IL-2 may enhance the anti-apoptotic properties of these cells in general, and such enhancement is effected by replicative senescence, at least in culture. On the other hand, it should be noted that down-regulation of specific genes, such as *SIVA1* (and probably many others) and up-regulation of genes encoding potent metastasis-associated factors in SEN hADSCs, is indicative of a troublesome phenomenon: IL-2 priming of senescent cells or exposure of the senescent cells to a pro-inflammatory environment might be critical in shifting the balance from immunomodulation to an environment promoting metastatic transformation and invasive growth.

### Transcriptional profiling suggests gene targets regulating enhanced migration and angiogenesis in IL-2 stimulated hADSCs upon replicative senescence

Further analysis of the transcriptional response indicates that IL-2 stimulation of senescent hADSCs profoundly enhances the expression of genes involved in vascular development and remodeling related to angiogenesis. We observed drastic up-regulation of the *VEGFA, VEGFB, FBLN5, FBLN7, PGF, ANGPT1, ANGPT2, ANGPTL2, ANGPTL6, TNFSF12, PRKCA, PIK3CA, HRAS* genes as well as a gene encoding a potent modulator of endothelial cell-leukocyte adhesion, *ESM1* (Figure 5D, Table 1 and Supplementary Table 1). The vascular endothelial growth factor, VEGF, released by MCSs enables recruitment of endothelial lineage cells and initiation of vascularization as was previously reported [43]. We have further demonstrated that up-regulation of *VEGFA* gene expression in SEN hADSCs can be detected by quantitative RT-PCR analysis and IL-2 priming results in a statistically significant increase of *VEGFA* gene transcription in SR and SEN hADSCs (Figure 6). Interestingly, we further observed that in response to IL-2 priming, a group of genes responsible for cell motility, migration and invasive growth are drastically up-regulated only in the hADSCs undergoing replicative senescence: *CGNL1, CGREF1, CRMP1, FGD6, TNK2, PTGS1, TNFAIP8, CTSB, CTSO, FAP, FERMT1, PLEKHA1, PLEKHA6, ROCK1, ROCK2*. In concert with this observation, our data indicate that a set of genes promoting cell adhesion, such as *CHD24, CYR61, ILK, NEDD9, MYL9, PPAP2B, RELN and TLN2* are down-regulated (Figure 5D and Supplementary Table 1). These data further support the experimental evidence for the enhanced migration capacity of senescent hADSCs shown in Figure 1C and Figure 1D.

Equally important, IL-2 priming results in the differential expression of a number of cytokines and factors critical for chemotaxis (shown in Figure 5B). For instance, SR hADSCs are marked by up-regulation of *IL-33, IL-12A, IL10RB, IL1RAP, IL7R, ILF2* and *NOS3* genes, while *IL-16* and *CSF1R* genes are down-regulated in these cells. In SEN hADSCs treated under similar conditions with IL-2, the genes encoding cytokines *IL-32, IL-6, IL1RN, IL20RB, IL21R* and inducers of inflammation *TNFSF13* and *TNFSF12,* as well as the gene encoding extracellular matrix remodeler *PLAU* are up-regulated (Table 1 and Supplementary Table 1). At the same time, several factors that are essential for cytokinesis such as *MYL9, KIF14, IRAC3,* as well as the gene encoding chemotactic factor that attracts monocytes and basophils (*CCL2)* and the *CLEC11A* gene regulating proliferation and differentiation of hematopoietic precursor cells, are down-regulated (Figure 5B). Similar down-regulation is also found for several interleukin receptor encoding genes *IL7R, IL1R1, IL15RA*, and interleukin enhancer binding factors *ILF2* and *ILF3*. Interestingly, *TNFSF13(APRIL),* a novel death-inducing secreted ligand, has been previously reported as both a cell proliferation-inducing [44] and cell death triggering ligand [45], thus cautioning that the role of the individual factors should be further investigated in a context-dependent manner.

All together, these data support the hypothesis that senescent mesenchymal stem cells, when subjected to inflammatory environment, might have decreased retention at the delivery site due to increased mobility. These same senescent cells may also play a role in tumor progression by promoting angiogenesis and metastasis. Our data also point to numerous biological targets critical for these events that can be prioritized for further experimental studies with *in-vivo* models and in clinical settings.

**Table 1:**
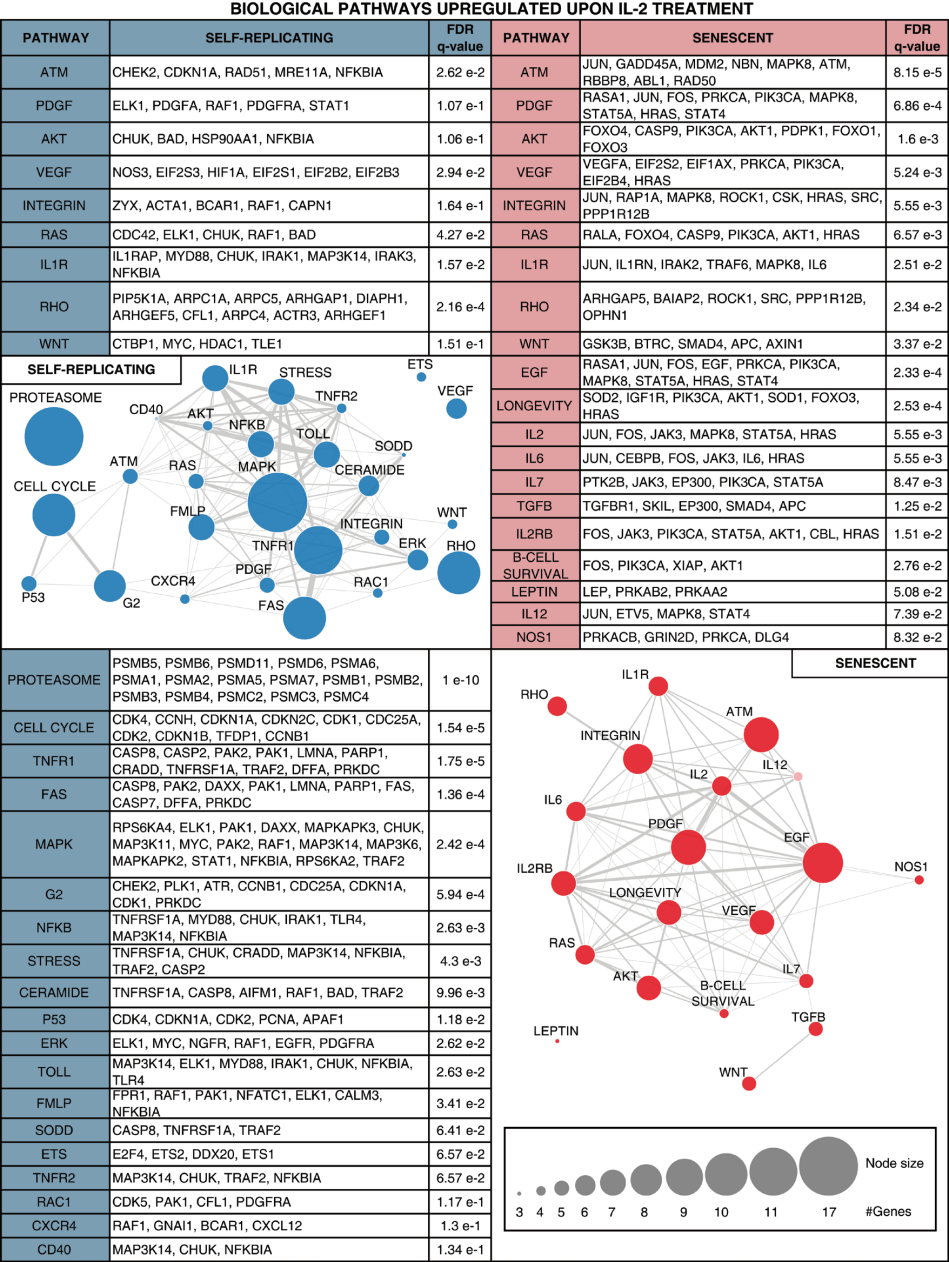
Biological pathways enriched for genes up-regulated upon IL-2 treatment in self-replicating (SR) and/or senescent (SEN) hADSCs

Enriched pathways are shown along with the individual IL-2+ up-regulated genes belonging to the pathway and the pathway enrichment significance levels. Pathways with gene members up-regulated in SR are shown in the left column, and pathways with gene members up-regulated in SEN are shown in the right column. Pathways with gene members up-regulated in both SR and SEN are shown in the top row followed by pathways with gene members up-regulated only in SEN, and finally pathways with gene members up-regulated only in SR. Networks are shown relating pathways that are up-regulated in SR (left column) and pathways that are up-regulated in SEN (right column). The network nodes represent pathways, and the sizes of the nodes correspond to the number of up-regulated genes in that pathway. Pathway nodes are connected by edges if the pathways share up-regulated genes, and edge-weights correspond to the number of up-regulated genes shared between the pathways.

## DISCUSSION

hADSCs are currently one of the primary sources of stem cells with direct clinical relevance [46]. Human MSCs may be able to both sense and respond to their immediate environment, which make these cells ideal to tune the response to injury and/or inflammation. It has been rightfully suggested that MSCs should be called Medicinal Signaling Cells as it was suggested in [47, 48].

The emerging evidence from studies of autoimmune disorders and models of tumorigenesis indicates that the immunomodulatory properties of adult mesenchymal stem cells can be susceptible to the presence of IL-2 in an inflammatory environment [49]. In addition, MSCs themselves can produce therapeutic cytokines, such as IFN-β [50] and interleukin-2 [51,52] to provide for beneficial anti-tumor effects. IL-2 is a potent cytokine that is also proven to boost the immune system to fight cancer. There are several FDA approved therapies currently on the market that make use of IL-2 to fight metastatic melanoma and renal cell carcinoma [53]. However, intravenous administration of IL-2 may have an impact on numerous tissues and organs in the body, including MSCs. Indeed, recent data indicate that MSCs in general, and MSCs derived from the adipose tissue, in particular, possess an intrinsic preferential property to migrate actively toward some tumor types upon their systemic administration [54-56]. What remains unclear is how adult mesenchymal stem cells may be affected by therapeutic doses of IL-2 and what, if any, changes in transcriptional outcomes occur with aging in response to IL-2 signaling. Our genome-wide transcriptomic analysis of hADSCS subjected to IL-2 exposure/priming *ex-vivo* demonstrates that senescence of stem cells substantially effects their transcriptional response.

IL-2 signals via specific receptors, with three classes of cell surface receptors formed by various combinations of three IL-2R subunits: IL2Rα (CD25), IL2Rβ (CD122) and IL2Rγ (CD132)[57]. Our experimental results indicate that hADSCs transcriptionally express all three receptors, however protein expression of the IL2Rα in hADSCs is far lower than seen for IL2Rβ. These observations suggest that an IL-2 receptor composition consisting of IL2Rβ and IL2Rγ isoforms might mediate the predominant form of IL-2 cytokine recognition by hADSCs. The receptor composition changes only slightly upon replicative aging of the hADSCs (Figure 2), indicating that responsiveness of hADSCs to IL-2 does not change upon their senescence. Alternatively, a slight increase in membrane-bound IL2Rα upon replicative senescence may reflect the activation state or ability to trans-present IL2Rα rather than responsiveness to IL-2, similar to what was previously reported for myeloid dendritic cells [58]. In our current study, we cannot discriminate between these events. In addition, our data also point out to the interesting phenomenon, that the production of IL2Rα drastically declines on transcriptional level with replicative senescence of adipose-derived MSCs. In accord with a previous report that in addition to cell surface IL-2Rα, IL-2Rα can exist in a soluble form (sIL-2Rα), which could be released from the cell surface [59], we speculate that our data might provide an indication of a less available soluble form of IL2Rα upon replicative aging of hADSCs.

In our current study, we also attempted to interrogate biochemical gene signaling networks activated in IL-2 primed SR and SEN hADSCs in order to gain insights into impact of the replicative senescence on adipose-derived MSCs function. Differential gene expression analysis, comparing IL-2 treated *versus* untreated SR and SEN cells, allowed us to uncover a number of individual genes that are up- or down-regulated upon IL-2 stimulation. However, the biological response to IL-2 treatment of hADSCs is not likely to be orchestrated by individual genes acting alone. Rather, this response is more likely to be based on multiple co-regulated genes that function together in integrated pathways. Furthermore, the biological pathways that are affected by IL-2 treatment are also likely to be functionally interconnected in the sense that they work together to execute cellular responses to stimulation by IL-2. We analyzed genes designated as up- or down-regulated in IL-2 treated SR and SEN hADSCs using an integrated gene-set enrichment and pathway network approach in an attempt to capture the biological reality of coordinated cellular responses to IL-2 stimulation. To do this, we first identified pathways that were statistically enriched for up- or down-regulated genes, and then we related these pathways based on the differentially expressed genes that they have in common (Table 1 and Table 2). We also weighted the pathway network representation based on the numbers of differentially expressed genes in each pathway and the extent to which different pathways share sets of differentially expressed genes. This approach allowed us to elucidate a highly connected network structure with numerous functionally related pathways as well as functionally relevant network substructures.

Notably, upon senescence of hADSCs IL-2 is less stimulatory for the gene pathways promoting proliferation (cell cycle pathway, *q-value = 1.54 e-5*), imposing G2 checkpoint (G2 pathway, *q-value = 5.94e-4*), p53 pathway (*q-value = 1.18e-2),* major signal transduction MAPK pathway (MAPK, *q-value = 2.42e-4*) and its major subgroup ERK pathway (ERK, *q-value = 2.62e-2),* which regulate important cellular function such as survival, migration and proliferation [60]. Our analysis further corroborates the previous finding that PDGF-induced AKT and ERK pathways regulate opposing fate decisions of proliferation and differentiation in order to promote MSC self-renewal [61]. Activation of the genes representing these pathways was observed only after *ex-vivo* IL-2 priming of actively self-renewing human ADSCs but not their senescent counterparts (Table 1, left side). Apparently, a different arm of the AKT pathway (including up-regulated *FOXO1, FOXO3* and *FOXO4* genes) acts upon exposure of SEN hADSCs to inflammatory environment (Table 1, right side), which could potentially be tumorigenic. The AKT pathway may be important to promote IL-6 secretion (both the gene and its biological pathway targets are transcriptionally up-regulated in senescence Table 1 and Figure 5B), which in turn has been shown to contribute to the establishment of the inflammatory environment and promote the resistance of fibroblasts to apoptosis [62-64].

Our data also provide insights into the functionality of MSCs in carcinogenic settings. Both SR and SEN hADSCs primed by IL-2 are marked by drastic increases in expression of transforming growth factors *alpha* and *beta* (*TGF*α*, TGF*β*1 and TGF*β*2*), transforming growth factor *beta* receptor *TGFBR2* and transforming growth factor *beta* receptor-associated protein *TGFBRAP1,* as well as transforming growth factor *beta*-induced (*TGFBI*) genes (Figure 5A). Secreted TGFβ is believed to be important in regulation of the immune system by promoting differentiation of CD4+ T-cells and inhibiting immune-surveillance, thereby imposing immunosuppression [65]. However, the higher level of TGFβ expression in hADSCs after exposure to IL-2 might promote carcinogenesis. Since parts of the TGFβ signaling pathway are shown to be mutated in cancer cells (see for the details in [66]), this allows cancer cells to escape TGFβ-induced cell cycle block, differentiation or apoptosis, while the surrounding stromal, immune, endothelial and smooth muscle cells still read the TGFβ signaling as a potent suppressor of proliferation and trigger of differentiation causing immunosuppression and angiogenesis in the cancer cell microenvironment. Cancer cells exploit this environmental condition to their advantage. In the absence of the effector T-cells, which normally attack cancer, cancer cells become more invasive [66-68]. Based on these observations, we speculate that therapeutic delivery of MSCs subjected either to prolonged expansion in culture, or into the patients burdened by chronic inflammation, are likely to create a microenvironment conducive to cancer progression and metastasis. Similarly, systemic administration of IL-2 as the treatment for cancer [69, 70] should take into consideration the age of the patient, since aging might diminish MSCs similar to what is seen for their *ex-vivo* replicative aging.

In support of this hypothesis, in IL-2 treated SEN hADSCs prominent up-regulated genes are enriched for pathways associated with inflammation (IL-6 pathway, *q-value = 5.55e-3*) and EGF signaling (*q-value = 2.33e-4)* that have been proven to provide a survival advantages to mesenchymal stem cells [71]. The SEN hADSCs primed with IL-2 are also marked by increased expression of *IL-1β*, *IL-6* and *IL-12* (Figure 5B), cytokines known to stimulate IL-17 from lymphocytes [72-74]. Our data also indicate that lymphocytes are the only source of IL-17D production, and those mesenchymal stem cells, particularly upon their senescence, display high transcriptional activity of IL-17 when subjected to a pro-inflammatory environment (Figure 5A). We hypothesize that the MCS-derived IL-17D together with MCS-derived CSF-1 might induce systemic neutrophil expansion and macrophages infiltration similar to studies indicating a critical role for these factors in promoting cancer progression and metastasis [75-77] as well as in a number of inflammatory diseases including psoriasis [73].

The observed connection to the angiogenic VEGF pathway (*q-value = 5.24e-3)* (Table 1, right side and Figure 5D) and the enhanced capacity of SEN hADSCs to migration (Figure 1C, 1D) may suggest that IL-2 primed SEN mesenchymal stem cells could acquire properties necessary to support a tumorigenic environment and metastasis. In addition, up-regulation of the genes included in nitric oxide synthase pathway (iNOS) NOS1 pathway (*q-value = 8.32e-2)* in hADSC upon replicative senescence once again support the hypothesis that mesenchymal stem cells undergoing senescence can acquire metastasis-promoting properties *via* immunosuppression.

In the current study, we further corroborated that aging poses a significant threat to adult MSC function [78-80] [7] by providing evidence that critical pathways essential for support of proliferation and DNA repair are down-regulated in hADCSs upon senescence: Cell Cycle pathway (*q-value = 2.52e-5),* MCM pathway *(q-value = 1.62e-8),* RB pathway *(q-value = 6.97e-5)* ATM pathway *(q-value = 3.28e-2),* p53 pathway *(q-value = 1.86 e-2)* shown in Table 2. Overall, our data indicate that there are more biological pathways subjected to IL-2 triggered down-regulation in senescence then in self-renewal and these biological pathways are interconnected (Table 2), further linking together a physiological impairment of IL-2 response upon replicative aging of hADSCs, thus suggesting that such impairment might be an integral to adipose-derived stem cell deviated function *in vivo* and upon clinical applications.

Our results, in concert with previously published data, should raise awareness that replicative aging of adult adipose-derived stem cells can impair their immunomodulatory, angiogenic and antioxidative functions, and in part, can explain several contradicting studies related to either tumor-promoting [81-83] or tumor-suppressive properties [55, 84] of adipose-derived MSCs. Our data also help to define biological pathways and gene targets for in depth exploration of functional activities of adipose-derived MSC therapeutic approaches and further refinement of their application in clinical settings.

**Table 2:**
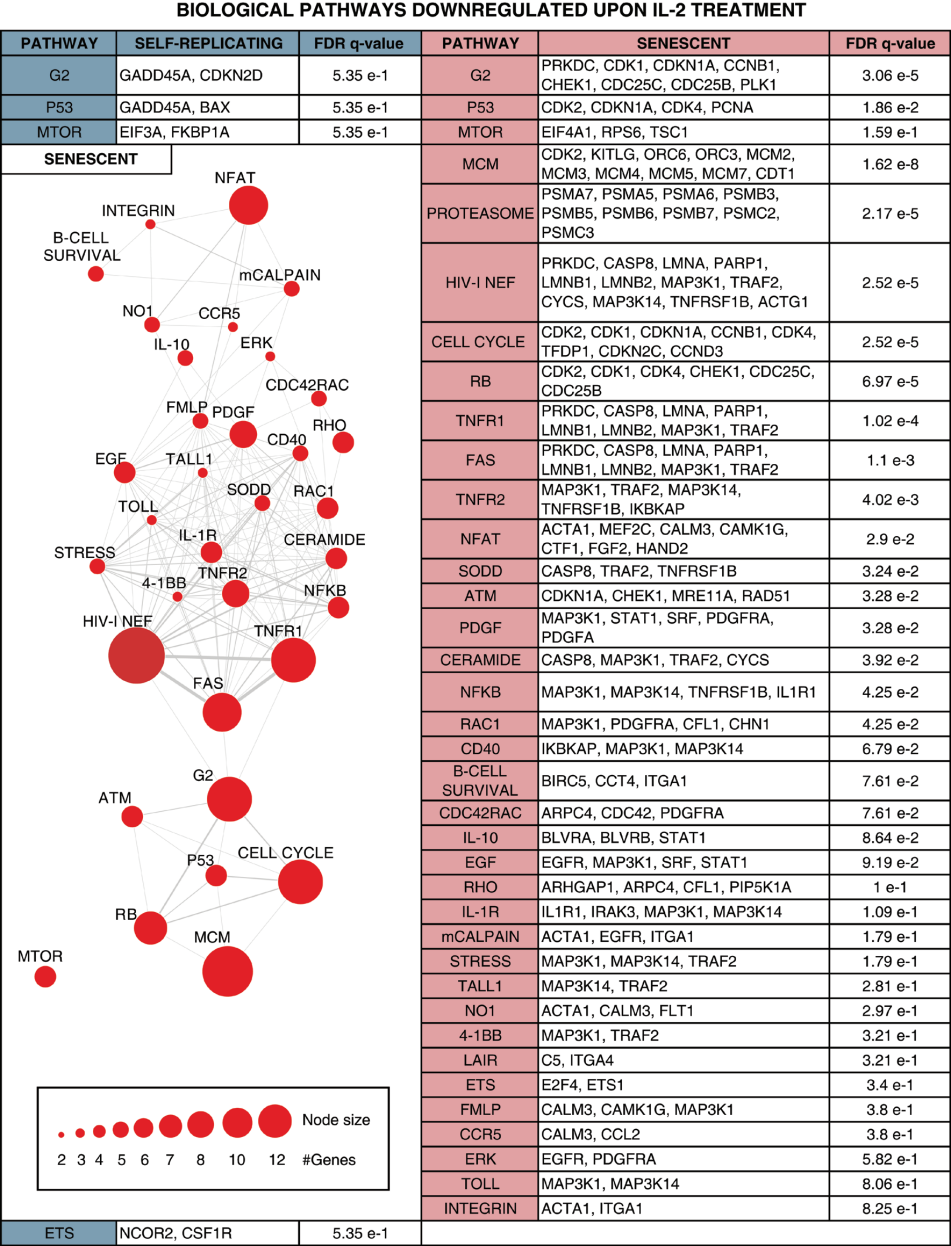
Biological pathways enriched for genes down-regulated upon IL-2 treatment in self-replicative (SR) and/or senescent (SEN) hADSCs

Enriched pathways are shown along with the individual IL-2+ down-regulated genes belonging to the pathway and the pathway enrichment significance levels. Pathways with gene members down-regulated in SR are shown in the left column, and pathways with gene members down-regulated in SEN are shown in the right column. Pathways with gene members down-regulated in both SR and SEN are shown in the top row followed by pathways with gene members down-regulated only in SEN, and finally pathways with gene members down-regulated only in SR. A network is shown relating pathways that are down-regulated in SEN (left column). The network nodes represent pathways, and the sizes of the nodes correspond to the number of SEN down-regulated genes in that pathway. Pathway nodes are connected by edges if the pathways share SEN down-regulated genes, and edge-weights correspond to the number of down-regulated genes shared between the pathways.

## MATERIALS AND METHODS

### Isolation, culture and characterization of MSCs

MSCs used in this research were isolated from human adipose tissues obtained from healthy adult female donors age 32 and 45 undergoing routine liposuction procedures at the UCSD medical center, San Diego, CA. The MSC isolation protocol was approved by the local ethics committee and performed as previously described [7]. Isolated adipose-derived stem cell lines were grown in DMEM/F12 medium (Life Technologies). In accordance with the MSC minimal definition criteria set by the International Society for Cellular Therapy [85], flow cytometric analysis showed that hADSCs express CD29, CD73, CD90 and CD105 but do not express CD11b, CD14, CD19, CD34, CD45, CD80, CD86 (antibodies from eBiosciense, USA). Morphological analysis showed that the cells present a fibroblast-like morphology, were plastic adherent and capable of adipogenic, chondrogenic and osteogenic differentiation under *in vitro* conditions using commercially available differentiation mediums (Invitrogen, USA). Cumulative population doublings (PD) were calculated as PD = log(N/N0) × 3.33 across the multiple passages as a function of the number of days of growth in culture as described in [7], where N0 is the number of cells plated in the flask and N is the number of cells harvested at this passage. hADSCs PD 4 or PD 6 for self-renewing populations (SR) and PD 41 and 38 for senescent populations (SEN) were used in all experiments. Treatment with recombinant IL-2 (Peprotech, USA) was performed as described in [86]. 20U/ml of IL-2 was added to the culturing media for 24 hours at 37°C.

### Senescence–associated SA-β galactosidase assay

The assay for monitoring the expression of pH-dependent senescence-associated β-galactosidase activity (SA-βGal) was performed as described in manufacturer's kit (BioVision) and previously published in [7]. The cultured hADSCs were fixed with fixative solution for 15 minutes at room temperature, washed with twice with PBS and stained with X-Gal containing supplement overnight at 37°C. The cells were washed twice with PBS, and the images were captured using a microscope (Nikons, TE300, DXM1200 Digital Camera, Japan).

### Migration and invasion assay

Transwell filters were from Corning Incorporated (Acton, MA, USA) and all the cytokines in use were obtained from Peprotech Inc. (Rocky Hill, NJ, USA). The migration assay was performed as described in [27] using 8mm thick Transwell chambers. For the Transwell migration assay, 1.0 × 10^4^ cells were suspended in 80μl of serum-free α-MEM and seeded in the upper chamber of 24-well Transwell plates containing 8 mm pore size filters (Corning, Costar, USA). In the lower chamber, 600 μl of DMEM or medium containing cytokines: IL-2, IL-6, IL-8, TNF-α, HMGβ1 was added. The concentrations used were: 50ng/ml IL-2, IL-6, IL-8 and HMGβ1; 30ng/ml TNF-α as described in [27]. hADSCs were incubated at 37°C for 16h. The cells retained in the upper chamber were removed by swab and those that had migrated through the filter were fixed with 4% paraformaldehyde for 20 minutes at room temperature and stained overnight with 5% toluidine blue. The cells were counted at the lower side; in five different randomly selected 10x fields using a bright-field microscope (NikonTE300, DXM1200 Digital Camera, Japan). These experiments were done with hADSCs of two female donors age 32 and 45, either self-renewing (SR) or senescent (SEN) populations, with each donor sampled more than three times.

### Enzyme-linked immunosorbent assays (ELISA)

hADSCs (SR or SEN) were plated at a density of 10^5^ cells per 10 cm^2^ dish and treated with 20 U/mL of IL-2 for 24 hour, with untreated controls as previously described in [86]. Then, cell membrane-associated protein fractions were prepared using Mem-PER Plus #89842 (ThermoFisher Scientific) following the manufacturer's protocol. Measurements of the concentrations of IL-2 receptors *alpha* and *beta* were obtained using human IL-2R *alpha* and human IL-2R *beta* ELISA kits #ELH-IL2Ra and #ELH-IL2Rb (RayBiotech, Inc) respectively. The optical densities for the standards (recombinant IL-2 receptors *alpha* and *beta)* as well as the experimental samples were measured at 450 nm by SPECTRA Max Plus (Molecular Devices) and concentrations were calculated as described in the manufacturer's protocol.

### Real-time quantitative polymerase chain reaction

Total RNA was isolated from hADSCs using the RNeasy Mini Kit (Qiagen, Germany). cDNA was then synthesized using the RevertAid First Strand cDNA Synthesis Kit (Fermentas, USA). Real-time quantitative polymerase chain reaction (qPCR) was performed using TaqMan instrument. The expression levels were calculated as 2−^ΔΔCt^, where relative expression was determined by normalization to *beta*-actin gene expression. All assays were conducted in triplicates and negative control samples without cDNA were used.

IL-2 Receptor Alpha chain (IL2Rα) For: 5′-CTGCCACTCGGAACACAAC-3′ and Rev: 5′-TGGTCCACTGGCTGCATT-3′.

IL-2 Receptor Beta chain (IL2Rβ) For: 5′-ACTCGAGAGCCAACATCTCC-3′ and Rev: 5′-TCCGAGGATCAGGTTGCAG-3′.

IL-2 Receptor Gamma 1 chain (IL2Rγ1) For: 5′-TGGATGGGCAGAAACGCTA-3′ and Rev: 5′-GGCTTCCAATGCAAACAGGA-3′.

STAT 5A For: 5′-ACGCAGGACACAGAGAATGA-3′

and Rev: 5′-CTGGGCAAACTGAGCTTGG-3′.

STAT 5B For: 5′-ACACAGCTCCAGAACACGT-3′ and

Rev: 5′-TGTTGGCTTCTCGGACCAA-3′.

VEGF A For: 5′-GGAGGAGGGCAGAATCATCA-3′ and

Rev: 5′-ATCAGGGGCACACAGGATG-3′.

### Transcriptomic analysis

Transcriptomic analysis was performed with IL-2 treated and untreated (control group) SR and SEN hADSCs as previously described in [86]. The two genotypes shown in Figure 1A were used for the analysis of four different conditions: self-renewal (SR), replicative senescence (SEN) without or with IL-2 stimulation respectively. Cells were seeded in DMEMF12 (10^6^) media for each experimental condition, and IL-2 treatment was performed by adding 20U/ml of recombinant IL-2 (Peprotech, USA) directly into the media for 24 hours as previously described in [86]. Total RNA was isolated from samples using TRIzol reagent (Invitrogen, USA) according to the manufacturer's instructions. Samples from two different patients were combined together for the relevant conditions and RNA concentrations were measured with the Qubit 2.0 fluorometer using the RNA HS Assay kit (Invitrogen, Life technologies, USA).

100ng of total RNA from each sample was used to construct the libraries for sequencing on the Ion Proton^TM^ System (Life technologies, USA) following manufacturers instructions. Prior to rRNA depletion and RNA-seq library construction, the ERCC RNA Spike-In Control mix (Ambion, Life Technologies) was added to total RNA for quality control analysis. The ERCC RNA Spike-In control mix contains 92 transcripts 250-2000 nt in length that mimic natural eukaryotic mRNAs. According to the protocol provided by manufacturer, for 100 ng of total RNA was added to 2ul of Mix1 in dilution 1:1000 of spike-in. Afterwards, rRNA depletion was performed with the Low Input Ribominus Eukaryote System v2 (Ambion, Life technologies, USA). cDNA libraries were constructed with Ion total RNA-seq kit v2 (Ambion, Life technologies, USA), and barcoded with Ion Xpress RNA-seq barcode (Ambion, Life technologies). The size distribution and quantification of the libraries were performed on a Bioanalyzer 2100 (Agilent technologies, USA). Library sequencing was performed on the Ion Proton^TM^ System with P1 chip, and each library was sequenced 3 times.

### RNA-seq data analysis

RNA-seq reads from individual Ion Proton^TM^ System sequencing runs were combined for each of the four libraries. Sequence reads were mapped to the reference human genome assembly hg19 (GRCh37) using the Torrent Mapping Alignment Program (TMAP, Life technologies). The quality of the four condition-specific combined RNA-seq runs was evaluated by comparing the expected counts of ERCC spike-in RNA sequences, obtained from the manufacturer's website, against the observed counts of RNA-seq tags that map to the same sequences (Supplementary Figure 2). Initial gene expression levels were taken as the sum of exon-mapped reads for individual NCBI RefSeq gene models (*c*), and lowly expressed genes (read counts per million < 1) were removed from subsequent analyses. For each library, individual gene expression levels were normalized using the beta-actin (*ACTB*) expression levels (*c_ACTB_*) and the total exon length *l* of each gene. For library *j*, the beta-acting normalization factor *s_j_* was calculated as $s_{j}=\frac{\frac{1}{n}\sum_{k=1}^{n}c_{ACTB,k}}{c_{ACTB,j}}$ and the final normalized expression value for gene *i* in library *j* was calculated as $e_{i,j}=\frac{c_{i,j}\times s_{j}}{l_{i}}$. Differential gene expression analysis between pairs of libraries was performed using the program GFOLD v1.1.3 [87]. GFOLD was chosen based on its demonstrated superior performance in characterizing differentially expressed genes in the absence of replicate data sets. GFOLD analysis yields a score that measures the extent of differential gene expression between conditions; the recommended GFOLD score cut-off of ±0.01 was used to define differentially expressed genes here. Functional enrichment analysis for differentially expressed genes between pairs of libraries was performed using the program GSEA v2.1.0 [88]. Specifically, individual pathways containing multiple genes that are up-regulated or down-regulated upon IL-2 treatment in SR, SEN or both were identified using GSEA. Individual pathways for specific sets of differentially regulated genes (IL-2+ up-regulated in SR and/or SEN and IL-2+ down-regulated in SR and/or SEN) were related using networks where the nodes correspond to pathways and the edges correspond to the presence of shared genes between pathways.

## SUPPLEEMENTARY MATERIAL FIGURES AND TABLE





## References

[1] Singer NG, Caplan AI (2011). Mesenchymal stem cells: mechanisms of inflammation. Annual review of pathology.

[2] Kim YY, Ku SY, Huh Y, Liu HC, Kim SH (2013). Anti-aging effects of vitamin C on human pluripotent stem cell-derived cardiomyocytes. Age (Dordr).

[3] Murphy MB, Moncivais K, Caplan AI (2013). Mesenchymal stem cells: environmentally responsive therapeutics for regenerative medicine. Exp Mol Med.

[4] Kuilman T, Michaloglou C, Mooi WJ, Peeper DS (2010). The essence of senescence. Genes Dev.

[5] Duggal S, Brinchmann JE (2011). Importance of serum source for the *in vitro* replicative senescence of human bone marrow derived mesenchymal stem cells. J Cell Physiol.

[6] Wagner W, Horn P, Castoldi M, Diehlmann A, Bork S (2008). Replicative senescence of mesenchymal stem cells: a continuous and organized process. PLoS One.

[7] Wang J, Geesman GJ, Hostikka SL, Atallah M, Blackwell B (2011). Inhibition of activated pericentromeric SINE/Alu repeat transcription in senescent human adult stem cells reinstates self-renewal. Cell cycle.

[8] Tollervey JR, Lunyak VV (2011). Adult stem cells: simply a tool for regenerative medicine or an additional piece in the puzzle of human aging?. Cell Cycle.

[9] Estrada JC, Torres Y, Benguria A, Dopazo A, Roche E (2013). Human mesenchymal stem cell-replicative senescence and oxidative stress are closely linked to aneuploidy. Cell Death Dis.

[10] O'Cearbhaill ED, Punchard MA, Murphy M, Barry FP, McHugh PE (2008). Response of mesenchymal stem cells to the biomechanical environment of the endothelium on a flexible tubular silicone substrate. Biomaterials.

[11] Bai L, Lennon DP, Caplan AI, DeChant A, Hecker J (2012). Hepatocyte growth factor mediates mesenchymal stem cell-induced recovery in multiple sclerosis models. Nat Neurosci.

[12] Holgate ST, Davies DE, Lackie PM, Wilson SJ, Puddicombe SM (2000). Epithelial-mesenchymal interactions in the pathogenesis of asthma. J Allergy Clin Immunol.

[13] Chen PM, Liu KJ, Hsu PJ, Wei CF, Bai CH (2014). Induction of immunomodulatory monocytes by human mesenchymal stem cell-derived hepatocyte growth factor through ERK1/2. J Leukoc Biol.

[14] Uccelli A, Moretta L, Pistoia V (2008). Mesenchymal stem cells in health and disease. Nat Rev Immunol.

[15] Ben-Ami E, Berrih-Aknin S, Miller A (2011). Mesenchymal stem cells as an immunomodulatory therapeutic strategy for autoimmune diseases. Autoimmun Rev.

[16] Ichim TE, Alexandrescu DT, Solano F, Lara F, Campion Rde N (2010). Mesenchymal stem cells as anti-inflammatories: implications for treatment of Duchenne muscular dystrophy. Cell Immunol.

[17] Yi T, Song SU (2012). Immunomodulatory properties of mesenchymal stem cells and their therapeutic applications. Arch Pharm Res.

[18] Ren G, Zhang L, Zhao X, Xu G, Zhang Y (2008). Mesenchymal stem cell-mediated immunosuppression occurs via concerted action of chemokines and nitric oxide. Cell Stem Cell.

[19] Glennie S, Soeiro I, Dyson PJ, Lam EW, Dazzi F (2005). Bone marrow mesenchymal stem cells induce division arrest anergy of activated T cells. Blood.

[20] Aggarwal S, Pittenger MF (2005). Human mesenchymal stem cells modulate allogeneic immune cell responses. Blood.

[21] Benvenuto F, Ferrari S, Gerdoni E, Gualandi F, Frassoni F (2007). Human mesenchymal stem cells promote survival of T cells in a quiescent state. Stem Cells.

[22] Xue Q, Luan XY, Gu YZ, Wu HY, Zhang GB (2010). The negative co-signaling molecule b7-h4 is expressed by human bone marrow-derived mesenchymal stem cells and mediates its T-cell modulatory activity. Stem Cells Dev.

[23] Zhu Y, Liu T, Song K, Fan X, Ma X (2008). Adipose-derived stem cell: a better stem cell than BMSC. Cell Biochem Funct.

[24] Puissant B, Barreau C, Bourin P, Clavel C, Corre J (2005). Immunomodulatory effect of human adipose tissue-derived adult stem cells: comparison with bone marrow mesenchymal stem cells. Br J Haematol.

[25] Yanez R, Lamana ML, Garcia-Castro J, Colmenero I, Ramirez M (2006). Adipose tissue-derived mesenchymal stem cells have *in vivo* immunosuppressive properties applicable for the control of the graft-versus-host disease. Stem Cells.

[26] Sohni A, Verfaillie CM (2013). Mesenchymal stem cells migration homing and tracking. Stem Cells Int.

[27] Perez LM, Bernal A, San Martin N, Galvez BG (2013). Obese-derived ASCs show impaired migration and angiogenesis properties. Arch Physiol Biochem.

[28] Galvez BG, San Martin N, Rodriguez C (2009). TNF-alpha is required for the attraction of mesenchymal precursors to white adipose tissue in Ob/ob mice. PLoS One.

[29] David D, Bani L, Moreau JL, Demaison C, Sun K (1998). Further analysis of interleukin-2 receptor subunit expression on the different human peripheral blood mononuclear cell subsets. Blood.

[30] Amu S, Gjertsson I, Brisslert M (2010). Functional characterization of murine CD25 expressing B cells. Scand J Immunol.

[31] Clausen J, Vergeiner B, Enk M, Petzer AL, Gastl G (2003). Functional significance of the activation-associated receptors CD25 and CD69 on human NK-cells and NK-like T-cells. Immunobiology.

[32] Simon HU, Plotz S, Simon D, Seitzer U, Braathen LR (2003). Interleukin-2 primes eosinophil degranulation in hypereosinophilia and Wells’ syndrome. Eur J Immunol.

[33] Lin JX, Leonard WJ (2000). The role of Stat5a and Stat5b in signaling by IL-2 family cytokines. Oncogene.

[34] Liao W, Lin JX, Wang L, Li P, Leonard WJ (2011). Modulation of cytokine receptors by IL-2 broadly regulates differentiation into helper T cell lineages. Nat Immunol.

[35] Mahmud SA, Manlove LS, Farrar MA (2013). Interleukin-2 and STAT5 in regulatory T cell development and function. JAKSTAT.

[36] Haynesworth SE, Baber MA, Caplan AI (1996). Cytokine expression by human marrow-derived mesenchymal progenitor cells *in vitro*: effects of dexamethasone and IL-1 alpha. J Cell Physiol.

[37] Cselenyak A, Pankotai E, Horvath EM, Kiss L, Lacza Z (2010). Mesenchymal stem cells rescue cardiomyoblasts from cell death in an *in vitro* ischemia model via direct cell-to-cell connections. BMC Cell Biol.

[38] Kim SY, Lee JH, Kim HJ, Park MK, Huh JW (2012). Mesenchymal stem cell-conditioned media recovers lung fibroblasts from cigarette smoke-induced damage. Am J Physiol Lung Cell Mol Physiol.

[39] Block GJ, Ohkouchi S, Fung F, Frenkel J, Gregory C (2009). Multipotent stromal cells are activated to reduce apoptosis in part by up-regulation and secretion of stanniocalcin-1. Stem Cells.

[40] Rehman J, Traktuev D, Li J, Merfeld-Clauss S, Temm-Grove CJ (2004). Secretion of angiogenic and antiapoptotic factors by human adipose stromal cells. Circulation.

[41] Py B, Slomianny C, Auberger P, Petit PX, Benichou S (2004). Siva-1 and an alternative splice form lacking the death domain, Siva-2, similarly induce apoptosis in T lymphocytes via a caspase-dependent mitochondrial pathway. J Immunol.

[42] Mei Y, Wu M (2012). Multifaceted functions of Siva-1: more than an Indian God of Destruction. Protein Cell.

[43] Chen L, Tredget EE, Wu PY, Wu Y (2008). Paracrine factors of mesenchymal stem cells recruit macrophages and endothelial lineage cells and enhance wound healing. PLoS One.

[44] Hahne M, Kataoka T, Schroter M, Hofmann K, Irmler M (1998). APRIL, a new ligand of the tumor necrosis factor family, stimulates tumor cell growth. J Exp Med.

[45] Kelly K, Manos E, Jensen G, Nadauld L, Jones DA (2000). APRIL/TRDL-1, a tumor necrosis factor-like ligand, stimulates cell death. Cancer Res.

[46] Kucerova L, Altanerova V, Matuskova M, Tyciakova S, Altaner C (2007). Adipose tissue-derived human mesenchymal stem cells mediated prodrug cancer gene therapy. Cancer Res.

[47] Caplan AI, Correa D (2011). The MSC: an injury drugstore. Cell Stem Cell.

[48] Caplan AI (2010). What's in a name?. Tissue Eng Part A.

[49] Ben-Ami E, Miller A, Berrih-Aknin S (2014). T cells from autoimmune patients display reduced sensitivity to immunoregulation by mesenchymal stem cells: role of IL-2. Autoimmun Rev.

[50] Studeny M, Marini FC, Champlin RE, Zompetta C, Fidler IJ (2002). Bone marrow-derived mesenchymal stem cells as vehicles for interferon-beta delivery into tumors. Cancer Res.

[51] Nakamura K, Ito Y, Kawano Y, Kurozumi K, Kobune M (2004). Antitumor effect of genetically engineered mesenchymal stem cells in a rat glioma model. Gene Ther.

[52] Stagg J, Lejeune L, Paquin A, Galipeau J (2004). Marrow stromal cells for interleukin-2 delivery in cancer immunotherapy. Hum Gene Ther.

[53] Schwartzentruber DJ (2001). Guidelines for the safe administration of high-dose interleukin-2. J Immunother.

[54] Nakamizo A, Marini F, Amano T, Khan A, Studeny M (2005). Human bone marrow-derived mesenchymal stem cells in the treatment of gliomas. Cancer Res.

[55] Khakoo AY, Pati S, Anderson SA, Reid W, Elshal MF (2006). Human mesenchymal stem cells exert potent antitumorigenic effects in a model of Kaposi's sarcoma. J Exp Med.

[56] Hung SC, Deng WP, Yang WK, Liu RS, Lee CC (2005). Mesenchymal stem cell targeting of microscopic tumors and tumor stroma development monitored by noninvasive *in vivo* positron emission tomography imaging. Clin Cancer Res.

[57] Robb RJ, Munck A, Smith KA (1981). T cell growth factor receptors. Quantitation, specificity, and biological relevance. J Exp Med.

[58] Driesen J, Popov A, Schultze JL (2008). CD25 as an immune regulatory molecule expressed on myeloid dendritic cells. Immunobiology.

[59] Rubin LA, Galli F, Greene WC, Nelson DL, Jay G (1990). The molecular basis for the generation of the human soluble interleukin 2 receptor. Cytokine.

[60] Rosova I, Dao M, Capoccia B, Link D, Nolta JA (2008). Hypoxic preconditioning results in increased motility and improved therapeutic potential of human mesenchymal stem cells. Stem Cells.

[61] Gharibi B, Ghuman MS, Hughes FJ (2012). Akt- and Erk-mediated regulation of proliferation and differentiation during PDGFRbeta-induced MSC self-renewal. J Cell Mol Med.

[62] Gehmert S, Wenzel C, Loibl M, Brockhoff G, Huber M (2014). Adipose tissue-derived stem cell secreted IGF-1 protects myoblasts from the negative effect of myostatin. Biomed Res Int.

[63] Mitsiades CS, Mitsiades N, Poulaki V, Schlossman R, Akiyama M (2002). Activation of NF-kappaB and up-regulation of intracellular anti-apoptotic proteins via the IGF-1/Akt signaling in human multiple myeloma cells: therapeutic implications. Oncogene.

[64] Gnecchi M, He H, Noiseux N, Liang OD, Zhang L (2006). Evidence supporting paracrine hypothesis for Akt-modified mesenchymal stem cell-mediated cardiac protection and functional improvement. FASEB J.

[65] Sheng J, Chen W, Zhu HJ (2015). The immune suppressive function of transforming growth factor-beta (TGF-beta) in human diseases. Growth Factors.

[66] Akhurst RJ, Derynck R (2001). TGF-beta signaling in cancer--a double-edged sword. Trends Cell Biol.

[67] Massague J (2008). TGFbeta in Cancer. Cell.

[68] Oskarsson T, Batlle E, Massague J (2014). Metastatic stem cells: sources, niches, and vital pathways. Cell Stem Cell.

[69] Payne R, Glenn L, Hoen H, Richards B, Smith JW (2014). Durable responses and reversible toxicity of high-dose interleukin-2 treatment of melanoma and renal cancer in a Community Hospital Biotherapy Program. J Immunother Cancer.

[70] Moore MJ (2007). Interleukin-2 in the treatment of unresectable or metastatic renal cell cancer: Time to write the obituary?. Can Urol Assoc J.

[71] Fan VH, Tamama K, Au A, Littrell R, Richardson LB (2007). Tethered epidermal growth factor provides a survival advantage to mesenchymal stem cells. Stem Cells.

[72] Chung Y, Chang SH, Martinez GJ, Yang XO, Nurieva R (2009). Critical regulation of early Th17 cell differentiation by interleukin-1 signaling. Immunity.

[73] Cai Y, Shen X, Ding C, Qi C, Li K (2011). Pivotal role of dermal IL-17-producing gammadelta T cells in skin inflammation. Immunity.

[74] Schwarzenberger P, Huang W, Ye P, Oliver P, Manuel M (2000). Requirement of endogenous stem cell factor and granulocyte-colony-stimulating factor for IL-17-mediated granulopoiesis. J Immunol.

[75] Chen WC, Lai YH, Chen HY, Guo HR, Su IJ (2013). Interleukin-17-producing cell infiltration in the breast cancer tumour microenvironment is a poor prognostic factor. Histopathology.

[76] Coffelt SB, Kersten K, Doornebal CW, Weiden J, Vrijland K (2015). IL-17-producing gammadelta T cells and neutrophils conspire to promote breast cancer metastasis. Nature.

[77] Escamilla J, Schokrpur S, Liu C, Priceman SJ, Moughon D (2015). CSF1 Receptor Targeting in Prostate Cancer Reverses Macrophage-Mediated Resistance to Androgen Blockade Therapy. Cancer Res.

[78] Demidenko ZN, Blagosklonny MV (2009). Quantifying pharmacologic suppression of cellular senescence: prevention of cellular hypertrophy versus preservation of proliferative potential. Aging (Albany NY).

[79] Bonab MM, Alimoghaddam K, Talebian F, Ghaffari SH, Ghavamzadeh A (2006). Aging of mesenchymal stem cell *in vitro*. BMC Cell Biol.

[80] Shiloh Y (2003). ATM and related protein kinases: safeguarding genome integrity. Nat Rev Cancer.

[81] Zimmerlin L, Donnenberg AD, Rubin JP, Basse P, Landreneau RJ (2011). Regenerative therapy and cancer: *in vitro* and *in vivo* studies of the interaction between adipose-derived stem cells and breast cancer cells from clinical isolates. Tissue Eng Part A.

[82] Prantl L, Muehlberg F, Navone NM, Song YH, Vykoukal J (2010). Adipose tissue-derived stem cells promote prostate tumor growth. Prostate.

[83] Yu JM, Jun ES, Bae YC, Jung JS (2008). Mesenchymal stem cells derived from human adipose tissues favor tumor cell growth *in vivo*. Stem Cells Dev.

[84] Grisendi G, Bussolari R, Cafarelli L, Petak I, Rasini V (2010). Adipose-derived mesenchymal stem cells as stable source of tumor necrosis factor-related apoptosis-inducing ligand delivery for cancer therapy. Cancer Res.

[85] Dominici M, Le Blanc K, Mueller I, Slaper-Cortenbach I, Marini F (2006). Minimal criteria for defining multipotent mesenchymal stromal cells. The International Society for Cellular Therapy position statement. Cytotherapy.

[86] Deenick EK, Gett AV, Hodgkin PD (2003). Stochastic model of T cell proliferation: a calculus revealing IL-2 regulation of precursor frequencies, cell cycle time, and survival. J Immunol.

[87] Feng J, Meyer CA, Wang Q, Liu JS, Shirley Liu X (2012). GFOLD: a generalized fold change for ranking differentially expressed genes from RNA-seq data. Bioinformatics.

[88] Subramanian A, Tamayo P, Mootha VK, Mukherjee S, Ebert BL (2005). Gene set enrichment analysis: a knowledge-based approach for interpreting genome-wide expression profiles. Proc Natl Acad Sci U S A.

