# Supplementary material for: Transcriptional profiling of interleukin-2-primed human adipose derived mesenchymal stem cells revealed dramatic changes in stem cells response imposed by replicative senescence
Source: Oncotarget. 2015 Jul 14;6(20):17938–57. doi: 10.18632/oncotarget.4852 (PMC4627227; doi:10.18632/oncotarget.4852)
Supplement: Supplementary file 1 [file oncotarget-06-17938-s001.pdf]

Transcriptional profiling of interleukin-2-primed human adipose derived mesenchymal stem cells revealed dramatic changes in stem cells response imposed by replicative senescence

Supplementary Material

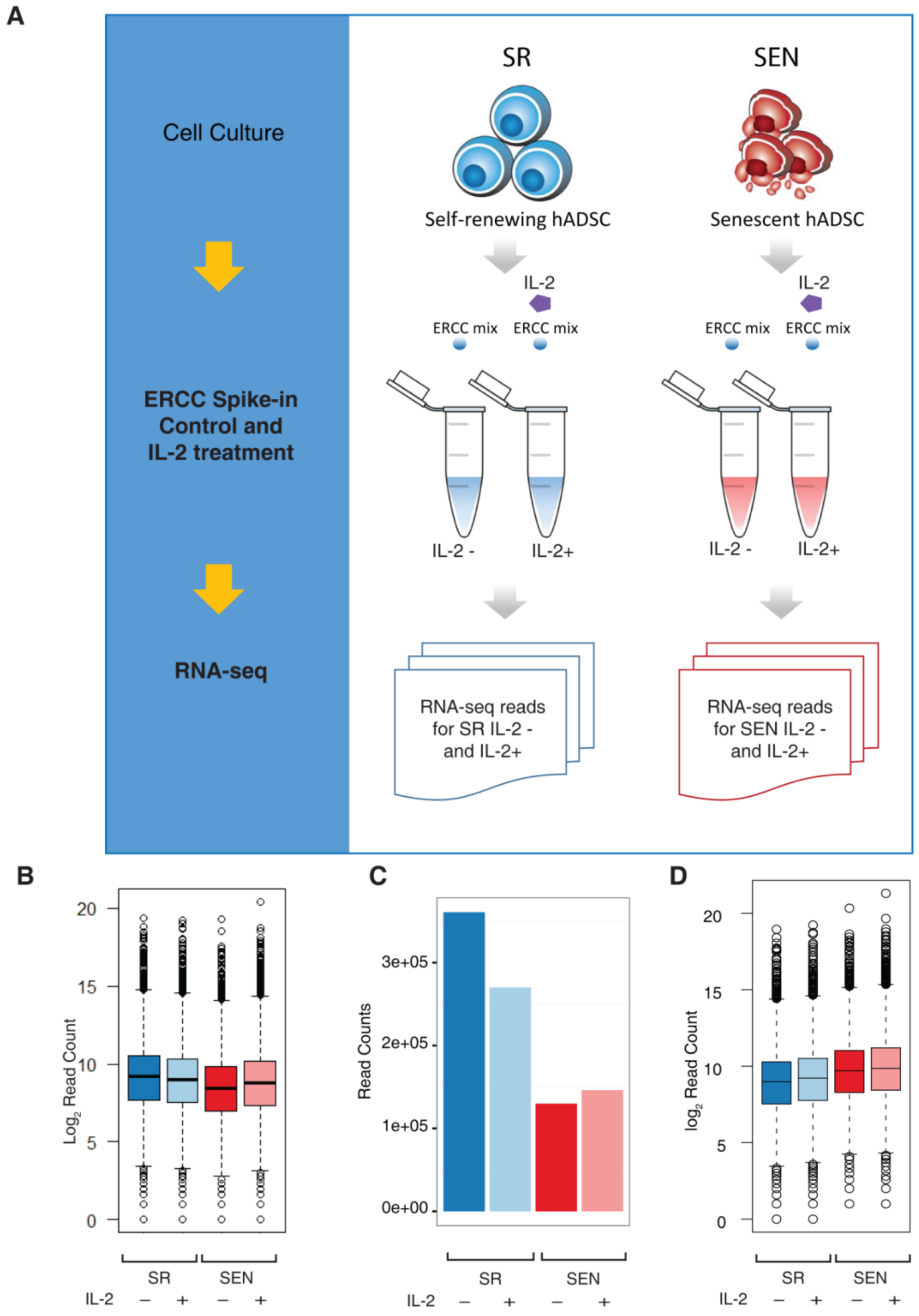

Supplementary Figure1. Experimental design and analysis for RNA-seq profiling of self-renewing and (SR) and senescent (SEN) hADSCs subjected to IL-2 treatment. (A)

Schematic representation of the RNA-seq experimental design. RNA was isolated from IL-2 treated and non-treated (control) SR and SEN cell culture samples and subject to library preparation and sequencing as described in the Materials and Methods. Panels B-D show the effect of the beta-actin (*ACTB*) normalization procedure used here (see Materials and Methods) on condition-specific RNA-seq gene expression levels. (B) Distributions of the gene-specific RNA-seq read counts for each condition prior to *ACTB* normalization. (C) Condition-specific RNA-seq read counts for *ACTB* that were used for normalization. (D) Distributions of the gene-specific RNA-seq read counts for each condition after *ACTB* normalization.

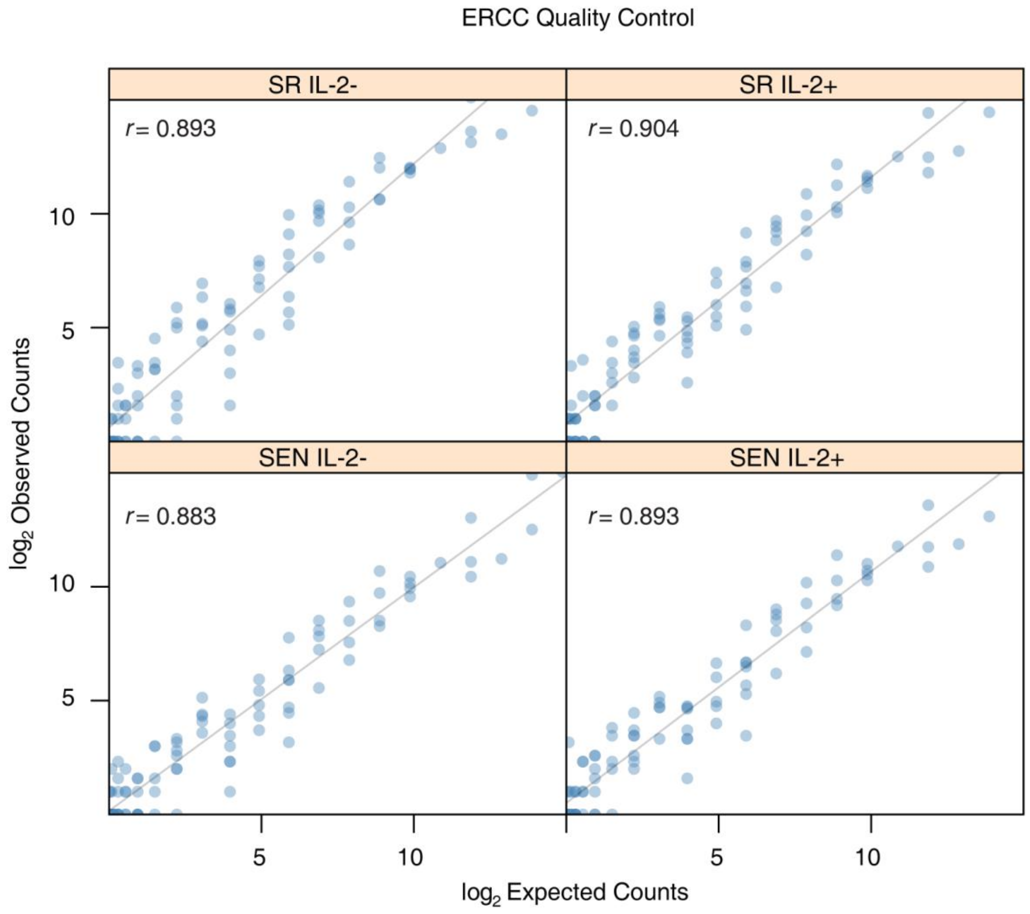

Supplementary Figure2. ERCC dose response used for quality control of RNA-seq experiments. For each of the four condition-specific RNA-seq pools, the expected counts of ERCC spike-in RNA sequences are regressed against the observed counts of RNA-seq tags that map to the same sequences. Observed versus expected counts are highly correlated, as indicated by the shape of the regression and the Pearson correlation *r*-values, consistent with high quality RNA-seq results.

Supplementary Table1. Table of the genes differentially expressed upon IL-2 treatment in self-renewing and senescence hADSCs .
